# A Spatially Distinct History of the Development of California Groundfish Fisheries

**DOI:** 10.1371/journal.pone.0099758

**Published:** 2014-06-26

**Authors:** Rebecca R. Miller, John C. Field, Jarrod A. Santora, Isaac D. Schroeder, David D. Huff, Meisha Key, Don E. Pearson, Alec D. MacCall

**Affiliations:** 1 Institute of Marine Sciences, University of California Santa Cruz, Santa Cruz, California, United States of America; 2 Fisheries Ecology Division, Southwest Fisheries Science Center, National Marine Fisheries Service, National Oceanic and Atmospheric Administration, Santa Cruz, California, United States of America; 3 Center for Stock Assessment Research, University of California Santa Cruz, Santa Cruz, California, United States of America; 4 Environmental Research Division, Southwest Fisheries Science Center, National Marine Fisheries Service, National Oceanic and Atmospheric Administration, Pacific Grove, California, United States of America; 5 California Department of Fish and Wildlife, c/o, National Marine Fisheries Service, Southwest Fisheries Science Center, Santa Cruz, California, United States of America; Hawaii Pacific University, United States of America

## Abstract

During the past century, commercial fisheries have expanded from small vessels fishing in shallow, coastal habitats to a broad suite of vessels and gears that fish virtually every marine habitat on the globe. Understanding how fisheries have developed in space and time is critical for interpreting and managing the response of ecosystems to the effects of fishing, however time series of spatially explicit data are typically rare. Recently, the 1933–1968 portion of the commercial catch dataset from the California Department of Fish and Wildlife was recovered and digitized, completing the full historical series for both commercial and recreational datasets from 1933–2010. These unique datasets include landing estimates at a coarse 10 by 10 minute “grid-block” spatial resolution and extends the entire length of coastal California up to 180 kilometers from shore. In this study, we focus on the catch history of groundfish which were mapped for each grid-block using the year at 50% cumulative catch and total historical catch per habitat area. We then constructed generalized linear models to quantify the relationship between spatiotemporal trends in groundfish catches, distance from ports, depth, percentage of days with wind speed over 15 knots, SST and ocean productivity. Our results indicate that over the history of these fisheries, catches have taken place in increasingly deeper habitat, at a greater distance from ports, and in increasingly inclement weather conditions. Understanding spatial development of groundfish fisheries and catches in California are critical for improving population models and for evaluating whether implicit stock assessment model assumptions of relative homogeneity of fisheries removals over time and space are reasonable. This newly reconstructed catch dataset and analysis provides a comprehensive appreciation for the development of groundfish fisheries with respect to commonly assumed trends of global fisheries patterns that are typically constrained by a lack of long-term spatial datasets.

## Introduction

Historical fisheries data provides insights into the development of fisheries activities and can inform efforts to interpret long-term trends, changes in species catch composition and the location of productive fishing habitats over time. Many fish populations worldwide undergo extraction activities where individual fishermen decide which species to pursue based upon market conditions and available gear technology [1], [2]. Selection of fishing grounds is determined from a variety of factors such as habitat, ocean productivity, distance to port and weather conditions [3]. Optimal fishing habitats are selected first where the expectation of catch is high and economic risks are low [4]. When catches become less reliable at previous fishing locations, harvest activities often shift to new habitats, leading to the geographic expansion of fisheries and fisheries impacts. Waters fished in the early years of the fishery may be associated with serial depletion in catch rates and species abundance [5], [6]. Both historical data and meta-analyses of global catch data suggest a large scale geographic expansion and serial depletion of fisheries across multiple stocks and regions [7]–[9], an increase in the spatial extent of the global fishing “footprint” [10], and a trend towards fishing greater depths over time [11], [12]. A number of empirical case studies have also documented patterns of fisheries development and consequent serial depletion of resources at regional scales for marine invertebrate populations [13], [14], [5], temperate water reef fishes [15], [16] shelf finfish species [17], [18], [6], coastal pelagic species [19], and deepwater seamount populations [20], [21]. Importantly however, geographic expansion is not necessarily synonymous with serial depletion and the effects of fisheries expansion are expected to vary by species, fishery and ecosystem [22].

The potential for geographic expansion and serial depletion are particularly problematic for evaluating fished populations in stock assessments, as it can lead to a perception of stability in catch size-structure and rates which could mask the true status of exploited populations [23], [24]. Most single-species stock assessments assume homogeneity in demographic structure and fishing mortality across space, and are typically incapable of explicitly addressing complex spatiotemporal patterns of fisheries development and/or spatial fisheries management [23]–[25]. While spatial assumptions in stock assessments are not likely to pose problems for stocks with high mixing or migratory behavior, other assessments of stocks with limited mobility, heterogeneity in population structure and/or distribution could be substantially flawed if incorrect assumptions regarding the behavior of the fishery are made, leading to bias in assessment results [25], [26]. Concerns over geographic expansion in California have been raised for several U.S. west coast groundfish stock assessments [27]–[29] and anecdotal accounts of serial depletion for some groundfish species provide evidence for its effect in California. For example, in a review of historical southern California fisheries, interviewed fishermen stated that historically vessels fishing with vertical line gear would “completely decimate” local rockfish *(Sebastes spp.)* concentrations in some habitats, facilitating a perpetual shift to new fishing locations that tended to be further offshore and at greater depths [30]. Regional-scale attempts to demonstrate geographic expansion found a similar transition in catch composition from shallow to deeper recreational rockfish species in Monterey Bay, California [31].

The goal of this study was to examine the spatiotemporal patterns and geographic expansion of commercial and recreational groundfish fisheries in California. We mapped the distribution and magnitude of groundfish catch by spatially summarizing the total catch per habitat area (CPA). For commercial groundfish, we map the year in which a given grid-block experienced catches that represented half of the cumulative total (Yr50) for species by preferred habitat depth stratum. We then used Generalized Linear Models (GLMs) to model Yr50 using variables that describe temporal patterns of historical fisheries development and oceanographic conditions over space including 1) distance from port, 2) depth, 3) the percent of days with wind speeds exceeding 15 knots (wind), 4) yearly mean chlorophyll-a (CHL) and 5) annual mean sea surface temperature (SST). The modeled geographic expansion for the entire state of California is the first of its kind and will provide a full picture of geographic expansion that will be useful in future stock assessments, catch reconstruction efforts, and marine spatial planning.

### California Groundfish Development

Rockfish, flatfish and other groundfish have been harvested commercially, recreationally and for subsistence in California waters for well over a century, with the earliest fisheries targeting nearshore and inland habitats, while later harvest expanded to more distant habitats. Native Americans first harvested groundfish for subsistence fisheries in nearshore reefs, collecting rockfish and other marine species when land-based food was in short supply [32]. Commercial fisheries activities in San Francisco Bay accelerated in the 1850s when the Gold Rush lured immigrants from around the globe. The immigrants brought new harvest techniques, such as the Italian paranzella (two-boat trawl), first introduced in 1876. By 1892 the port of San Francisco contributed 93% of reported catch for the entire state of California [33], with much of the fishing activities taking place in the Bay and Sacramento River. However, depletion concerns over inshore groundfish stocks led area closures of trawl nets for some species in San Francisco Bay in 1906 [34] and waters off southern California in 1913 [35] forcing vessels to pursue fish habitats further from ports. In order to keep track of fish harvest and sales a monthly fish ticket system, also known as landing receipts, was enacted in 1911 by California Department of Fish and Wildlife (CDFW, previously California Department of Fish and Game). Each ticket recorded the species or market category, pounds caught, date, and price. Further ticket refinements in 1933 included a spatial-catch “grid-block” system which was implemented out of necessity because distances traveled to fish no longer reflected the locality of landing port [36]. In order to travel to more distant harvest grounds, fishing vessels switched from gasoline motors to diesel engines and increased capacity with ice and refrigeration. The need for inexpensive protein and vitamin A from shark livers to support the war effort led to a sharp increase in trawl effort and landings, particularly in northern California waters after 1941 [37]. After a slight post-WWII decline in landings the groundfish fishery diversified and included a wide range of rockfish, flatfish and roundfish species. Many of these species were previously harvested only by hook and line, due to greater depths and rough habitats, but by 1940s and 1950s were targeted by otter trawl gear. The 1950s ushered in a new generation eager for recreational fisheries leisure; fiberglass rod and reels allowed anglers to easily fish deep-water rockfishes, with some ports reporting a 400–500% increase over an 8-year span [38]. Many recreational fishers either used their own personal watercraft or paid a fare to board a chartered commercial passenger fishing vessel (CPFV) also known as “Party Boats.” Recreational fisheries continue to be a significant proportion of total catch throughout the state, representing the majority of total catch for many nearshore and shelf species, particularly throughout the Southern California Bight (SCB) [39]. Globalization of the 1960s–1970s lured foreign factory trawlers to California where they harvested rockfish and other groundfish populations offshore, outside of what was then considered to be a three nautical-mile territorial sea. Foreign vessel rockfish catches were substantial, with approximately 50,000 tons harvested in California [40], nearly 35% of the domestic rockfish catch during this period. To ensure that coastal resources were harvested domestically rather than by foreign vessels, national policies encouraged rapid growth of the US West Coast groundfish fleet and banned foreign vessels to the 200 mile limit off the US coastline [41], [42]. During this period ships became evermore hardy and the ability to fish and travel in rough weather was less of a constraint. In the 1980s development of midwater species, such as widow rockfish *(S. entomelas)*, also led to new fishing opportunities, but yet again, rapid depletion of the newly developed resources often followed [43]. Increasing vulnerability concerns of the fishery resource relative to fishing power constrained harvest levels and landings quotas in the 1990s as stock assessments began to recognize the low productivity, longevity, and vulnerability of rockfish populations to overexploitation [44]–[46]. In the early 2000s, seven rockfish species were declared to be overfished, and efforts to rebuild populations resulted in tremendous reductions in allowable catches of most rockfish species, and other co-occurring groundfish species, for both commercial and recreational fisheries [44], [47]. Efforts to rebuild populations led to the implementation of a substantial network of spatial areas closed to groundfish fishing [48], [49], the largest being the nearly 11,000 km^2^ Cowcod Conservation Area (CCA) in the SCB. The boundary of the CCA was established based on observation of higher relative abundance in this area when the stock was declared overfished in 2001 [50]. Consequently, the future management system for West Coast groundfish is likely to require spatially explicit management for some time.

## Methods

### California Groundfish Spatial Datasets

**Figure 1:**
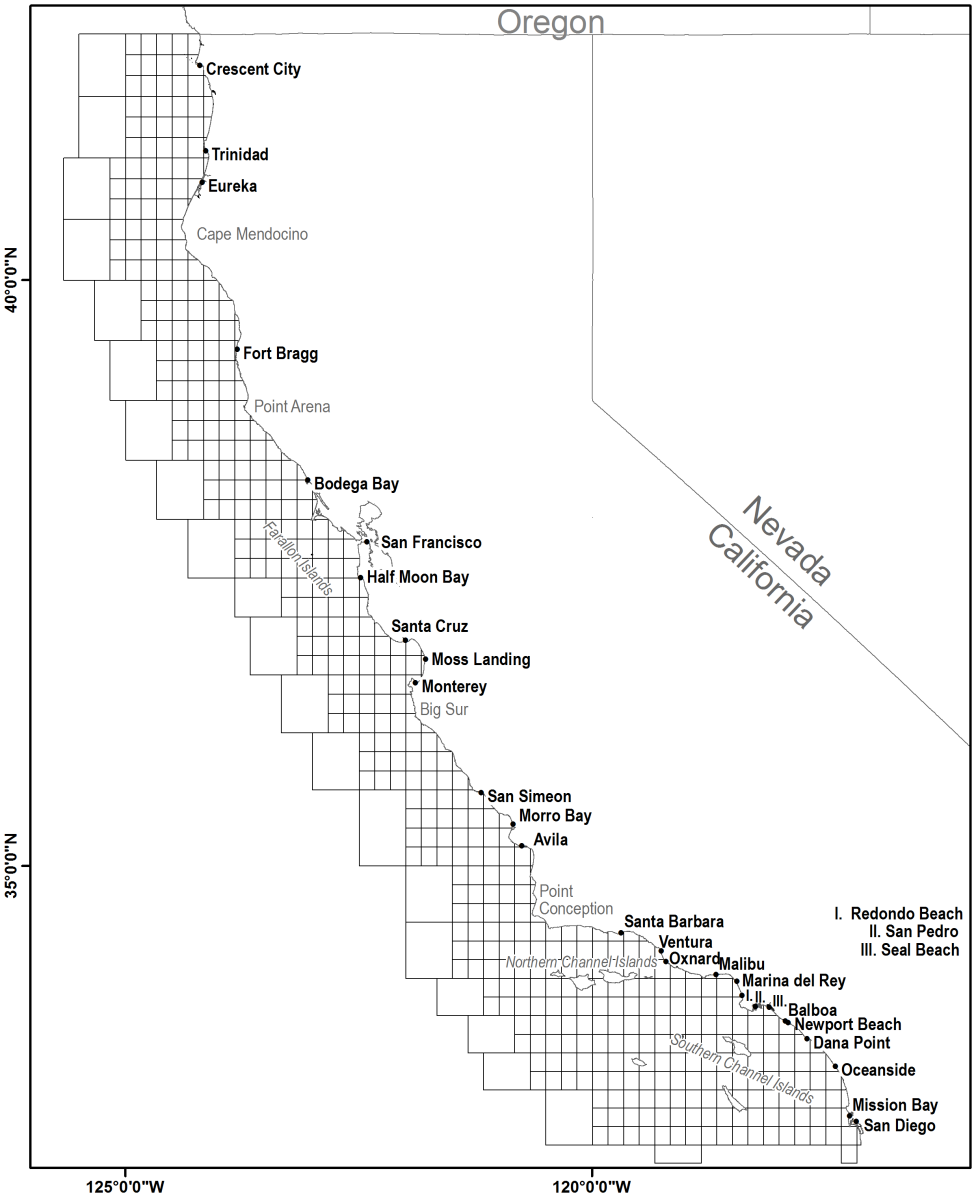
California Department of Fish and Game spatial blocks established in the 1930s. The commercial and recreational ports accounting for 95% of total catch are labeled in black. Geographic features are labeled gray.

A unique set of spatially referenced, long-term commercial groundfish catch data from 1933–1968 were recently recovered and digitized from microfiche records as part of an ongoing catch reconstruction effort between the National Marine Fisheries Service (NMFS) and CDFW [49]. In 1933 the CDFW established a spatially distinct “grid-block” data collection system where gridded cells representing ocean habitat were partitioned into 554 blocks at a 10×10 minute grid resolution [51], [52] (Figure 1). When commercial fishermen bring fish to port a landing receipt is produced. This receipt holds detailed information on species type, pounds caught, date, price and spatial block [53]. The record keeping in general is detailed, however information such as gear type and effort were not consistently available, therefore gear-specific and effort analyses are not possible for the early period of the fishery. Landing receipts from 1969–2010 were queried from the California Cooperative Survey (CalCOM) database [54] using both trawl and non-trawl gear types. As the data used here reflect the landings data from fish tickets, they were analyzed with respect to market categories, rather than species-specific estimates. Table 1 lists the species and market categories that accounted for virtually all groundfish landings analyzed, including the fraction of the total catch accounted for by each market category over the time period evaluated. As most groundfish spend the majority of their adult life stage near the ocean bottom, species and market categories were assembled into representative depth strata that best approximated their greatest population densities, as a proxy for habitat. The depth strata included were 0–200 m, 0–400 m, 0–600 m or 100–1200 m. For most species, the market categories are relatively “pure” to the species level, however the rockfish market categories in particular typically reflect a broad (and non-stationary) grouping of species that required considerable analysis to “reconstruct” back to species-level catches [51]. With the exception of Pacific halibut (*Hippoglossus stenolepis)*, managed by the International Pacific Halibut Commission, and California halibut (*Paralichthys californicus)*, managed by the CDFW, virtually all groundfish species in this study are managed under the Pacific Fishery Management Council’s Groundfish Fishery Management Plan [50], adopted in 1982. The recreational rockfish block summary data from CPFV’s were archived in a CDFW dataset beginning in 1936 in southern California and 1957 in central and northern California, and is based on self-reported numbers of fish landed (i.e. vessel operator logbooks), as documented in Hill and Schneider [55]. Rockfish were seldom reported to the species level before the 1980s, and only sporadically thereafter, so we lumped all recreational rockfish into a single *Sebastes spp*. category. As with the commercial fishery, over 50 species of rockfish are either commonly or infrequently encountered in recreational fisheries. The most commonly encountered species include blue (*Sebastes mystinus*), black (*S. melanops*), vermillion (*S. miniatus*), bocaccio (*S. paucispinis*), olive (*S. serranoides*), chilipepper (*S. goodei*), yellowtail (*S. flavidus*) and canary (*S. pinniger*) rockfish [51]. Catches are reported as number of fish kept.

**Table 1:** Evaluated commercial groundfish market categories and the fraction of the total catch from 1935–2000.

Common name	Scientific name	% of total
0 to 200 meters depth		
English sole	*Parophrys vetulus*	7.14%
Sanddab, Pacific, Speckled, other	*Citharichthys sordidus, C. stigmaeus*	1.91%
California Halibut	*Paralichthys californicus*	1.77%
Starry Flounder	*Platichthys stellatus*	1.06%
Skate, Big, California, other	*Raja binoculata, R. inornata*	0.73%
Sole, Rock, Butter, other	*Lepidopsetta bilineata, Isopsetta isolepis*	0.40%
Sand sole	*Psettichthys melanostictus*	0.26%
California Scorpionfish	*Scorpaena guttata*	0.17%
Leopard shark	*Triakis semifasciata*	0.11%
Turbot, Curlfin, Hornyhead, other	*Pleuronichthys decurrens, P. verticalis*	0.11%
Cabezon	*Scorpaenichthys marmoratus*	0.10%
Kelp Greenling	*Hexagrammos decagrammus*	0.00%
Sculpin, Staghorn, Yellowchin, other	*Leptocottus armatus, Icelinus quadriseriatus*	0.00%
0 to 400 meters depth		
Pacific Whiting	*Merluccius productus*	5.54%
Petrale Sole	*Eopsetta jordani*	4.72%
Lingcod	*Ophiodon elongatus*	3.07%
Arrowtooth Flounder	*Atheresthes stomias*	0.25%
Pacific Halibut	*Hippoglossus stenolepis*	0.07%
Pacific Cod	*Gadus macrocephalus*	0.00%
Pacific Tomcod	*Microgadus proximus*	0.00%
Slender Sole	*Lyopsetta exilis*	0.00%
0 to 600 meters depth		
Unspecified rockfish	*Sebastes spp.*	21.61%
Bocaccio rockfish	*Sebastes paucispinis*	4.56%
Widow rockfish	*Sebastes entomelas*	3.04%
Chilipepper rockfish	*Sebastes goodei*	1.46%
Splitnose rockfish	*Sebastes diploproa*	0.50%
Yellowtail rockfish	*Sebastes flavidus*	0.44%
100 to 1200 meters depth		
Dover sole	*Microstomus pacificus*	20.03%
Sablefish	*Anoplopoma fimbria*	11.73%
Thornyheads, Longspine, Shortspine	*Sebastolobus alascanus, S. altivelis*	5.72%
Rex sole	*Glyptocephalus zachirus*	2.73%
Spiny Dogfish shark	*Squalus acanthias*	0.60%
Grenadiers, Giant, Pacific, other	*Coryphaenoides acrolepis, Albatrossia pectoralis*	0.19%

Species are grouped into habitat depth strata that best approximated their greatest population densities.

**Figure 2:**
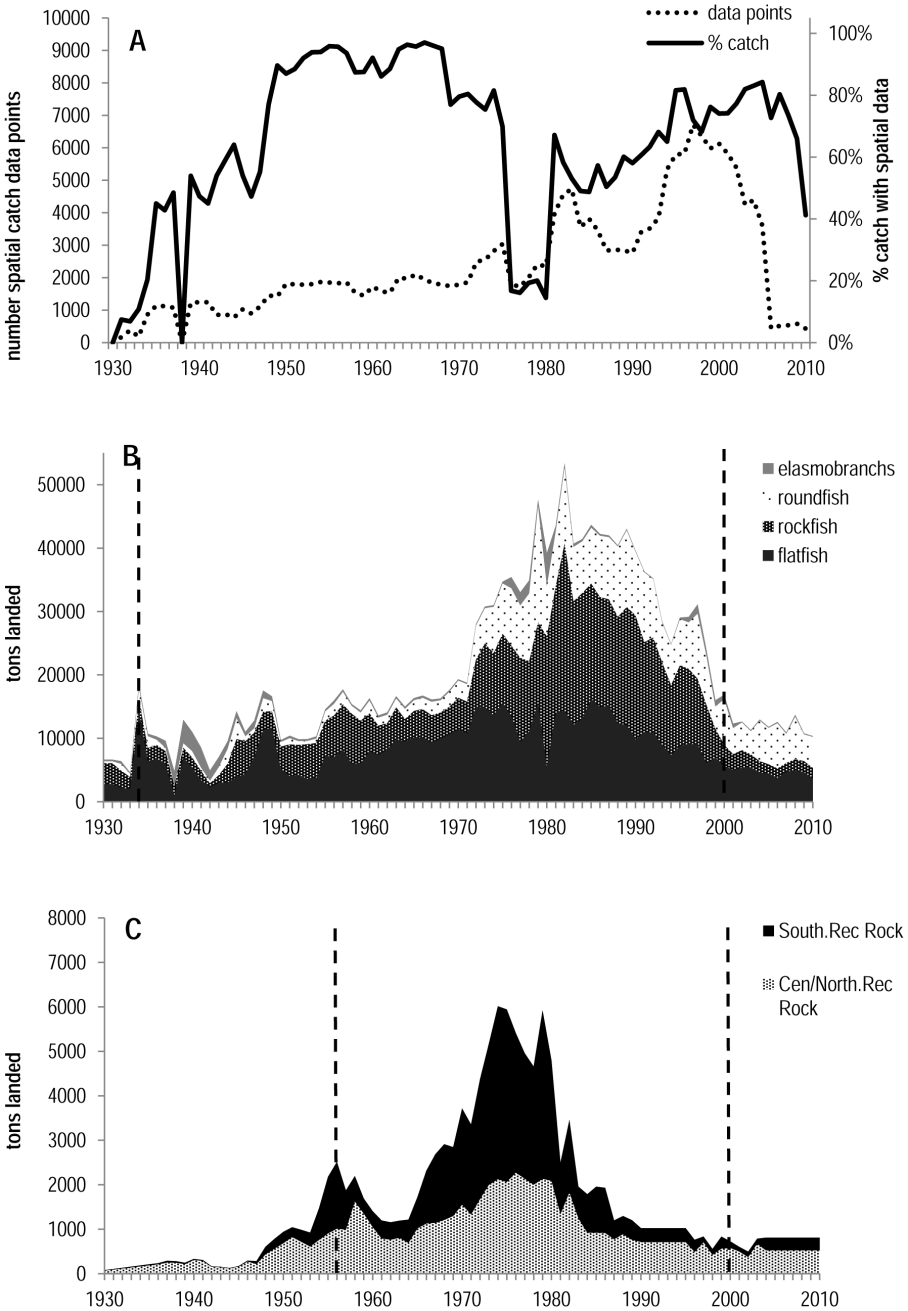
California groundfish landing receipts from 1930 –**2010.** Figure 2a shows the total number of observations for commercial fisheries and the percentage of the estimated total catch associated with spatially distinct block information. Figure 2b shows the total California commercial groundfish landings in metric tons for the 1930–2010 period. Dashed line indicates begin and end year selected for analysis. Figure 2c shows the total estimated metric tons of recreational rockfish landed for the 1930–2010 period (from [51]). Dashed line indicates begin and end year selected for analysis.

Historical reported catches were reviewed and adjusted to address gaps in spatial block reporting. Although spatial block reporting to CDFW was technically mandatory, not all of the catches included block-specific information. The first several years of this program were associated with very low reporting rates, however an increase in reporting rates occurred by the mid–1930s and by the 1950s–1975 approximately 80–100% of the commercial catch was associated with spatial information (Figure 2a) (see [51] for details). This was the basis for the decision to begin our analysis of commercial data in 1935, when approximately 50% of catches had plausible spatially-explicit catch information. Reporting requirements on landing receipts were relaxed in the 1970s and 1980s and the rate of block reporting declined, however landings associated at the regional scale and trawl logbook data collection remained mandatory. Spatial data for recreational rockfish were not archived in all California regions until 1957, therefore for consistency purposes we chose a start date of 1957. While recreational catches prior to 1957 are not trivial, catches were modest relative to those from 1957–2000. An end date of 2000 was used for both commercial and recreational fisheries because spatial management measures enacted at that time had substantial impacts on both magnitude of catches and ability of fishermen to select habitats [49]. The catch for some grid-blocks were considered implausible because the blocks include habitat beyond the depth range of the reported species (e.g. rockfish species reported in depths greater than 800 meters, at which very few individuals in this genus are encountered [56], [57]). This is likely a consequence of at least two factors; intentional misreporting in which fishermen deliberately reported the wrong statistical area of the catch, and inadvertent misreporting in which fishermen may have fished multiple species in different locations on a single trip but only included a single block on their landing receipt. To address these concerns, we removed blocks in which catches were highly unlikely to occur and scaled the catches within the remaining blocks such that the sum of the year-specific landings equaled the commercial landings estimates by groundfish species category; rockfish, flatfish, roundfish, and elasmobranchs (Figure 2b and Figure 2c). Historical catches from foreign fishing vessels were excluded, due to lack of spatial information and the fact that catches from these at-sea fisheries operations would not be related to proximity to ports. Consequently, this analysis focuses on the total magnitude of the catch and the temporal pattern of fisheries development, rather than an analysis of spatially explicit catch rates.

### Mapping Groundfish Yr50 and CPA Values

For each grid-block, we spatially mapped the values of geographic expansion and total historical catch for groundfish species by preferred habitat depths. The spatiotemporal development of California groundfish catch history is described as the year at 50% cumulative historical catch (Yr50). The Yr50 is the year in which half of the total reported catch within a given block was reported, representing the temporal element of geographic expansion for these fisheries. By necessity, the previously described depth strata for each species or market category were simplified to reflect the majority distribution of a given species where fishing occurs, despite the fact that some species may occur infrequently over shallower or greater depths. A 1200 meter cutoff was based on the Pacific Fishery Management Council’s (PFMC) Essential Fish Habitat (EFH) analysis which found historical fishing effort rarely extended past 1200 meters and usage of trawl gear is currently prohibited in depths greater than 1280 meters [58]. Recreational rockfish were assembled into a single 0–200 m depth stratum, as most recreational fishermen rarely fish deeper than 200 m. Blocks were stratified by depth based upon the U.S. Coastal Relief Model bathymetry data, which is the best available seamless bathymetry dataset containing all blocks at the highest resolution, 3-arc seconds (90m) [59]. The total catch per area (CPA) was calculated as metric tons per block habitat (km^2^) for all evaluated years (respective to each fishery type). For commercial groundfish we map CPA for each depth stratum and spatially summarize. Blocks containing less than 1 km^2^ habitat were removed in order to reduce potential CPA over-inflation biases associated with small areas. CPA provides the relative magnitude of cumulative removals relative to total available habitat and represents a combined consequence of exploitation and productivity of a given depth-defined habitat. Yr50 and CPA values were calculated for each fishery for all depth strata and mapped using an equal quantile distribution, where the 10 classification breaks represents each subsequent 10th percentile.

### Modeling Geographic Expansion of Groundfish

GLM covariates were developed for each block, representing oceanographic and habitat conditions that fishermen might consider before selecting fishing habitats (i.e. blocks). Habitat covariates were calculated for each depth stratified block, where a centroid was considered a representative location since it is unknown where within each block groundfish species were caught. The minimum Euclidian distance between habitat centroids and ports was calculated. Port landings for groundfish are unavailable prior to 1969, so to be consistent we used ports which received 95% of the cumulative rockfish catch during the period 1969–2000. Since only a small number of ports met this criterion in northern California, we included the top 4 ranked ports in this region, as the port of Eureka comprised a large proportion of the groundfish catch. Mean bathymetry per block was calculated using the Zonal Statistics tool (Spatial Analyst, ESRI ArcMAP v.10 [60].

For each block we developed an annual mean of SST, CHL and % days with high winds using satellite data over 10 years (1999–2009). SST, CHL, and wind speed were derived from Pathfinder, SeaWiFS, and QuikSCAT satellites data products, respectively [61]–[63] and all data products were downloaded from NOAA CoastWatch (http://coastwatch.pfeg.noaa.gov/erddap/). Spatial subsets and monthly means of SST (day and night) and CHL were used in the creation of the annual means to improve spatial coverage due to missing data from cloud cover [64]. We selected percentage of days during 1999–2009 with wind magnitudes greater than 15 knots as an indicator of how likely a given habitat may have been fishable, under the assumption that winds greater than 15 knots are likely to be constraining to effective fishing operations. Bilinear interpolation was used to place the satellite annual means onto the CDFW grid. QuikSCAT is unable to measure nearshore winds resulting in only 381 of the 554 blocks with wind estimates. These missing blocks were filled by using nearest neighbor interpolation or merged with nearshore buoy data (NDBC bouys and M1 mooring in Monterey Bay). To maintain data normality, we arcsin square-root transformed wind percentages and log-transformed chlorophyll-a values for analysis purposes.

### Analysis

variable Yr50 to identify which combinations of explanatory covariates best explain commercial groundfish and recreational rockfish geographic expansion. In order to reduce collinearity we calculated a Pearson’s correlation coefficient for all potential explanatory covariate pairs, where a high correlation was considered >0.6. We found that SST was highly correlated to wind (r = 0.73 commercial and r = −0.69 recreational) and logCHL was highly correlated to distance from port (r = −0.62 commercial). Therefore, we removed SST and logCHL from the list of potential commercial fisheries explanatory variables and SST from the list of potential recreational variables in order to avoid distortion in model estimation [65]. Each candidate model was fitted using R software [66]. For model selection, we chose a single model with the lowest Akaike Information Criterion (AIC) score [67] if it was allocated most of the AIC weight (MuMln package [68]). We plotted the response of each model covariate across the range of data values while holding the other model covariates at their median values [69]. We mapped the model predictions based on sample covariates from each geographic block (MGET [70]).

## Results

### Mapping Groundfish Yr50 and CPA Values

**Figure 3:**
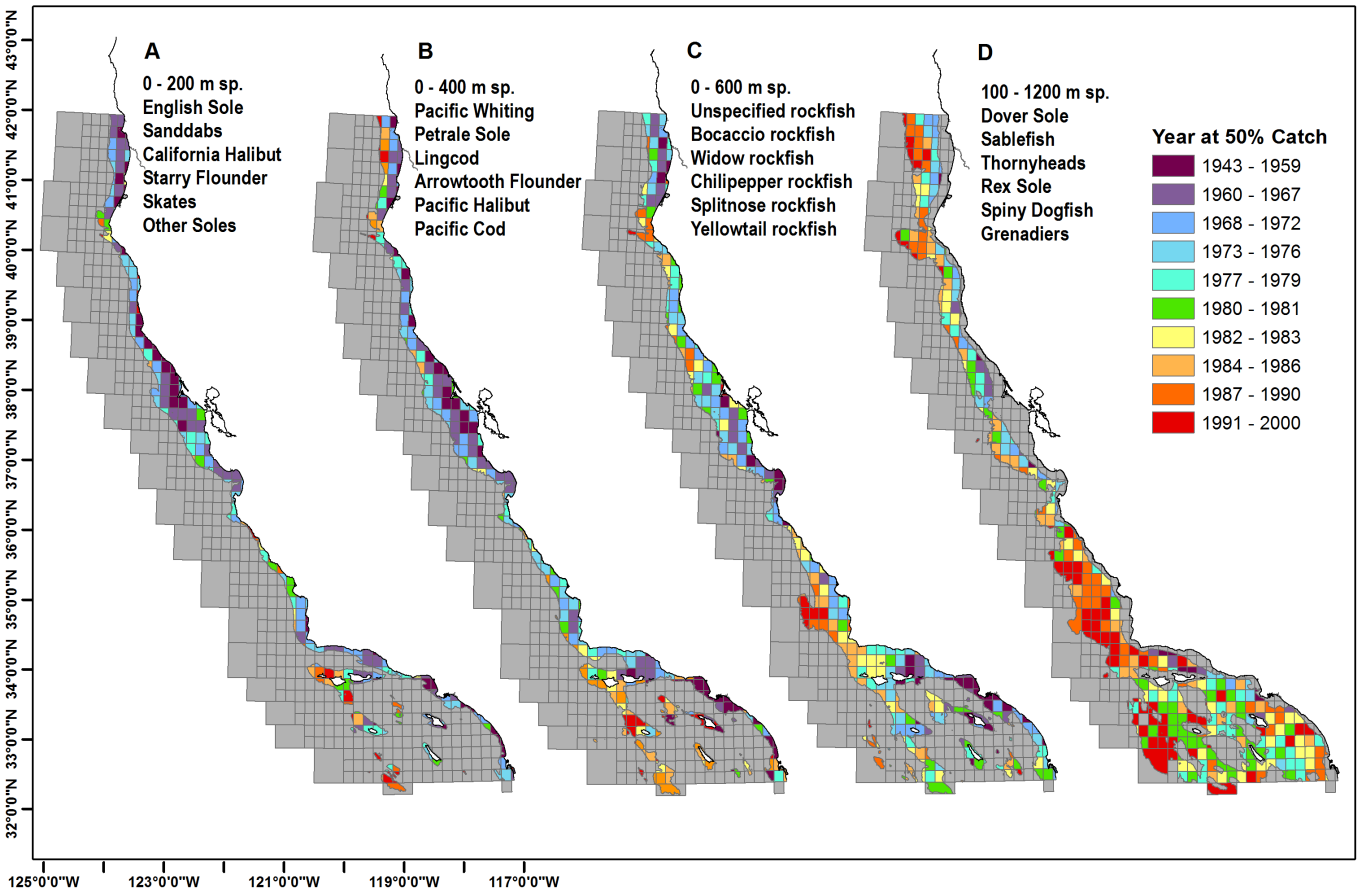
Year at 50% cumulative catch (Yr50) values by block for commercial groundfish. Spatial blocks are stratified by depth strata representing habitat of each groundfish species and include Fisheries Management Plan (FMP) species and a subset of state-managed species that are generally landed or encountered with FMP species. Named species are top 6 by landing weight. 3a. Yr50 values for 0–200 m species. 3b. Yr50 values for 0–400 m species. 3c. Yr50 values for 0–600 m species. 3d. Yr50 values for 100–1200 m species. Yr50 classifications are based on an equal quantile distribution.

The blocks for commercial species, bounded by depth strata representing habitat, demonstrate shallower species were caught earlier than deep water species (Figure 3a–d). For some blocks the Yr50 occurred as early as 1943, while the latest occurred in the year 2000. The 200 m and 400 m species had an earlier Yr50 catch near San Francisco and Eureka, and later Yr50 catch near the offshore Channel Islands. The 600 m species, all rockfish, have earlier catches near Eureka, Monterey, San Pedro and the nearshore Channel Islands, while the later catches occurred near Cape Mendocino and the Santa Lucia Bank, west of Avila. The 100–1200 m species have many blocks where Yr50 occurred after 1976, and blocks concentrated west of Avila, Cape Mendocino and Crescent City have a Yr50 during the 1980s–2000s.

**Figure 4:**
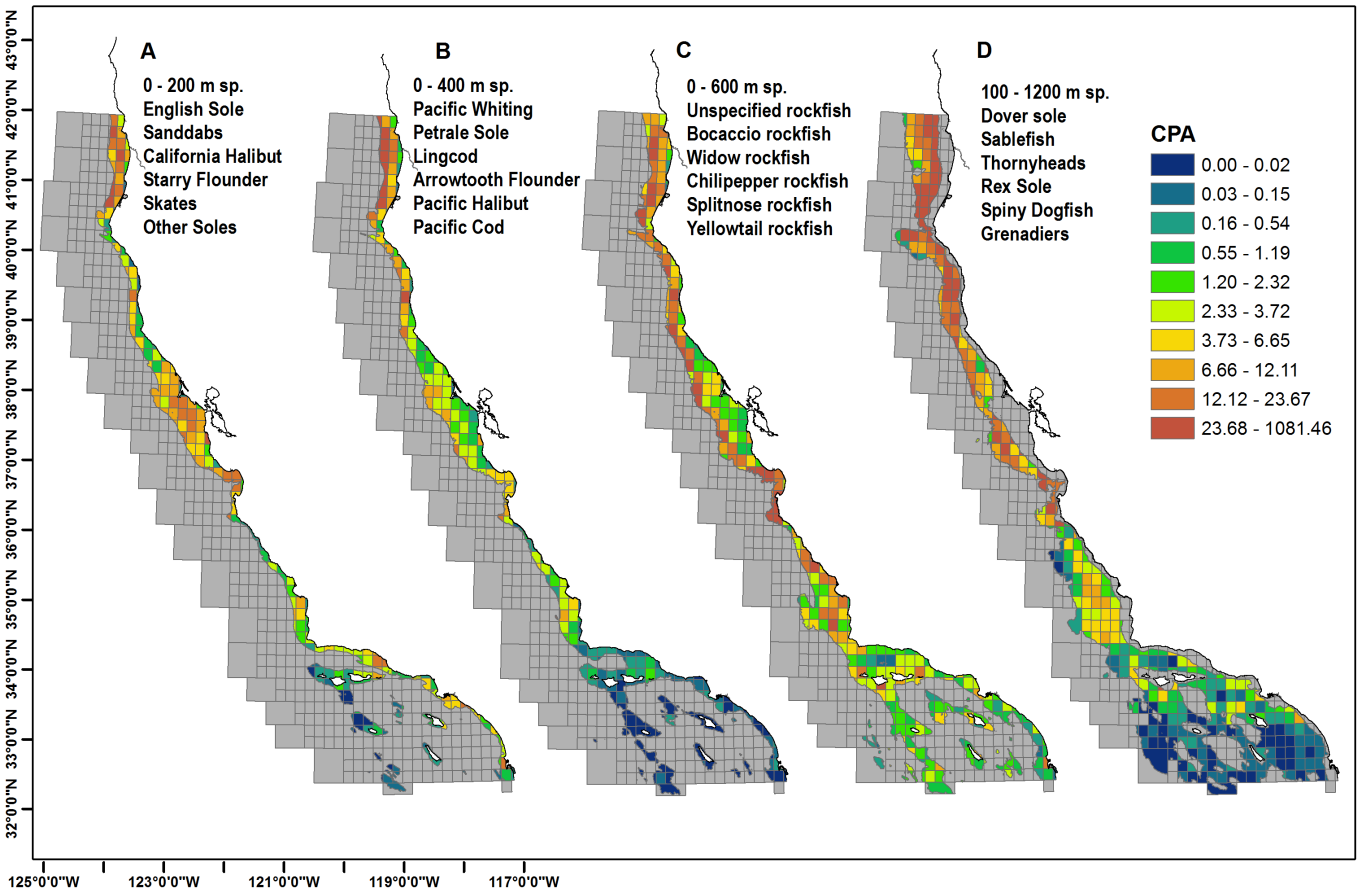
Commercial groundfish total Catch per Area (CPA) in metric tons per block habitat (km^2^) for the years 1935 –**2000.** Spatial blocks are stratified by depth strata representing habitat of each groundfish species and include Fisheries Management Plan (FMP) species and a subset of state-managed species that are generally landed or encountered with FMP species. Named species are top 6 by landing weight. Total Catch is reported in tons. 4a. CPA for 0–200 m species. 4b. CPA for 0–400 m species. 4c. CPA for 0–600 m species. 4d. CPA for 100–1200 m species. Yr50 classifications are based on an equal quantile distribution.

The historical total CPA for the shallow water commercial (0–200 m) species, including California Halibut, Greenlings (Hexagrammidae) and Sanddabs (*Citharichthys spp.)*, tended to be located near Crescent City and Eureka, and shallow sandy areas outside of San Francisco Bay (Figure 4a). The 0–400 m species, including Pacific Whiting *(Merluccius productus)* and Petrale Sole (*Eopsetta jordani)*, had the largest catches near the shelf breaks just west of Crescent City and Eureka (Figure 4b). The 0–600 m species, all rockfish spp., were concentrated on the shelf breaks from northern California to Morro Bay (Figure 4c). The deepwater species (100–1200 m) largely sablefish (*Anoplopoma fimbria)*, dover sole (*Microstomus pacificus)* and thornyheads (*Sebastolobus spp.*), were concentrated throughout Northern California and Monterey Bay, while a moderate amount was caught west of Avila (Figure 4d).

**Figure 5:**
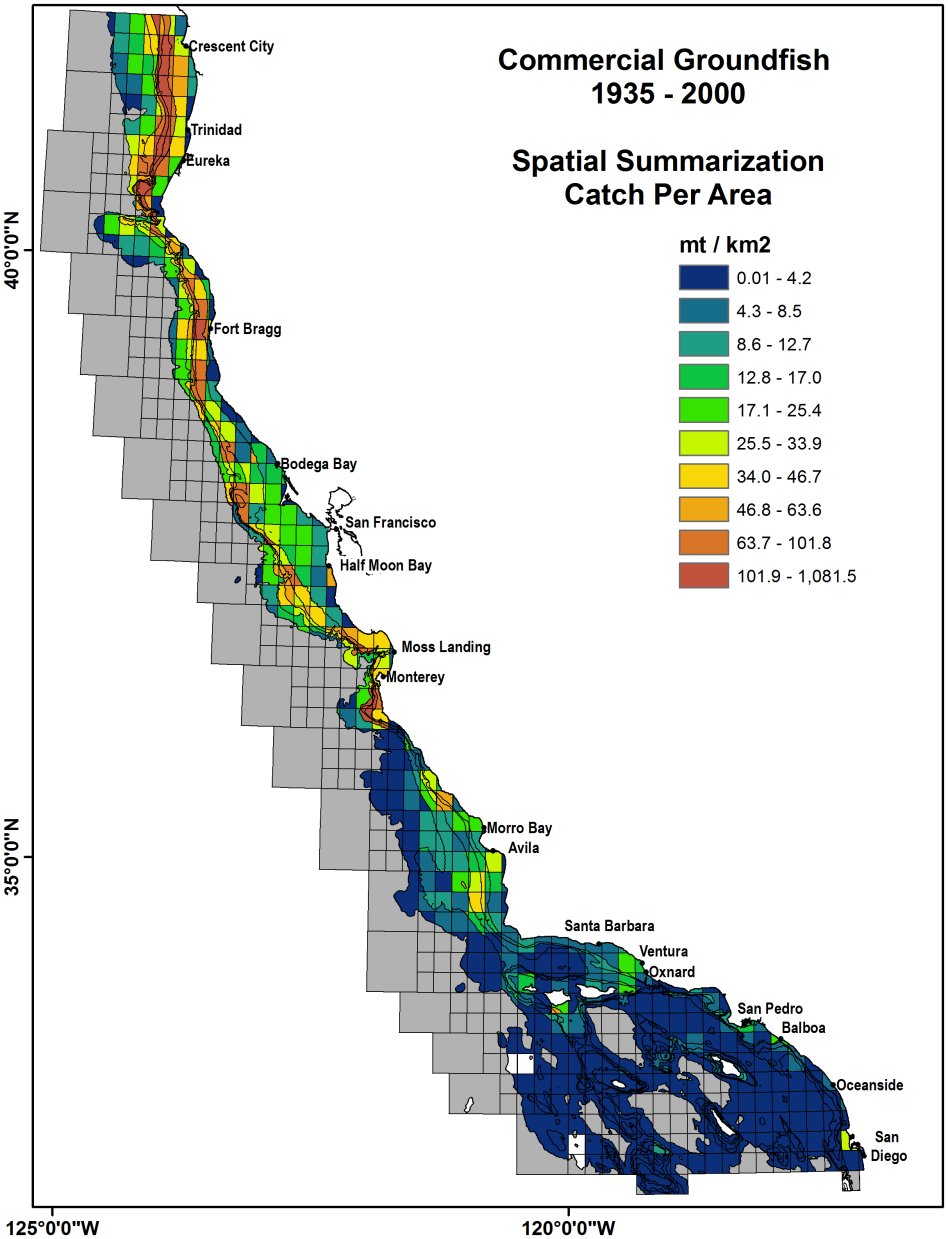
Spatial summarization of commercial groundfish total Catch per Area (CPA) in metric tons per block habitat (km^2^) for the years 1935 –**2000.** Gridded overlay represents catch blocks stratified by depth strata representing habitat of each groundfish species, 200 m, 400 m, 600 m, 1200 m. Ports shown are identified as the top 95% commercial ports by total catch.

**Figure 6:**
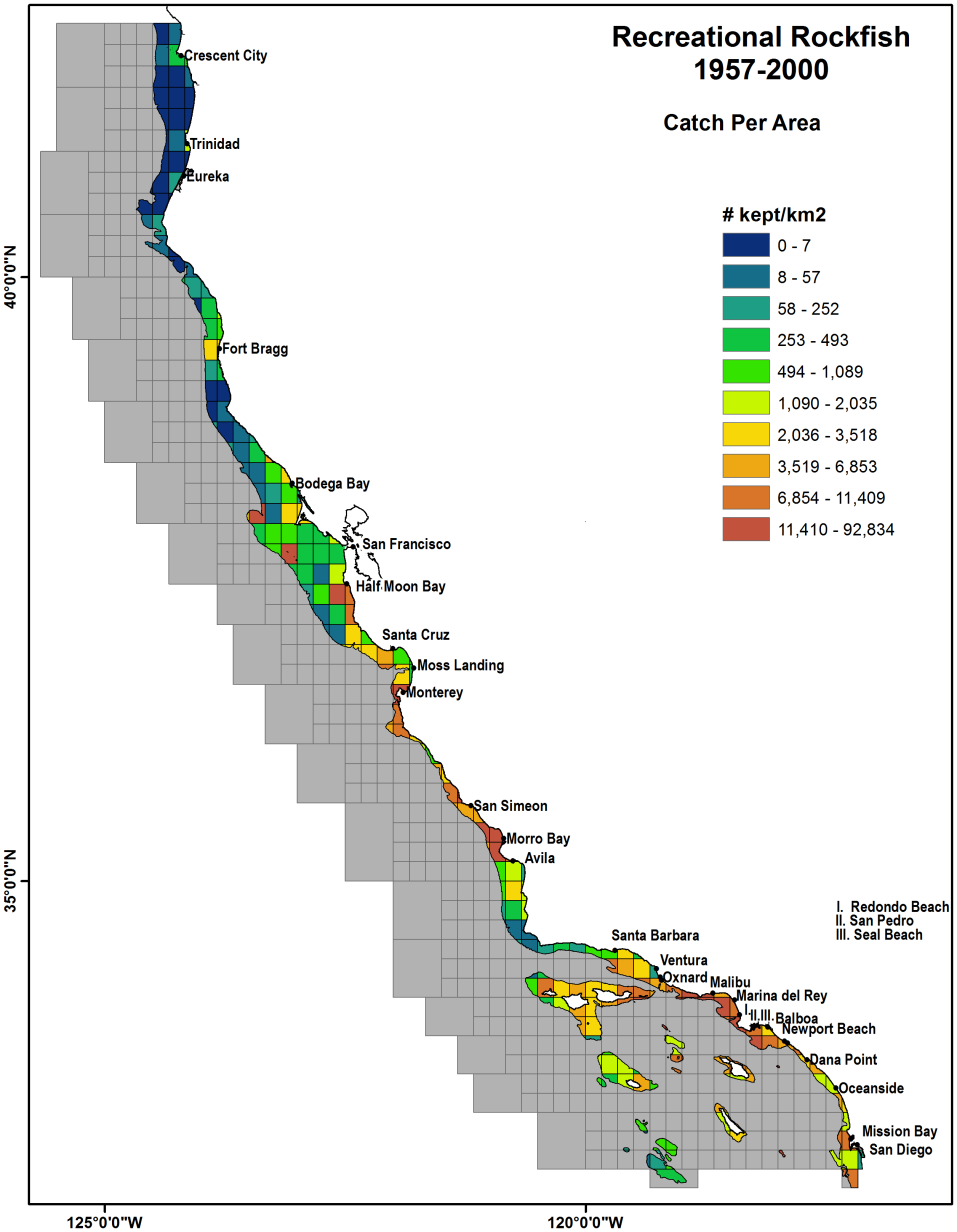
Spatial summarization of recreational rockfish total Catch per Area (CPA) in number of fish kept per block habitat (km^2^) for the years 1957 –**2000.** Gridded overlay represents catch blocks stratified by 200 m depth. Ports shown are identified as the top 95% recreational ports by total catch.

The spatially summarized CPA for commercial groundfish had the largest catches near the continental shelf breaks in northern and central California while CPA for recreational fisheries was located at inshore blocks near the SCB, Morro Bay and Monterey (Figure 5 and 6). The summarized CPA values for all commercial groundfish were concentrated in the Northern and Central California continental shelf breaks and were much greater than in the SCB. Recreational rockfish CPA values show large catches for inshore blocks in Southern California near Redondo Beach, Terminal Island and at many of the off-shore islands of Santa Catalina, Santa Cruz, Santa Barbara and San Clemente. In the central coast, large recreational catches occurred near Morro Bay, Monterey and Half Moon Bay, along with the offshore sites of Cordell Bank and Farallon Islands (Figure 6). Blocks along the northern California coast had a smaller recreational rockfish CPA with respect to southern and central California.

### Modeling Geographic Expansion (Yr50) of Groundfish

**Figure 7:**
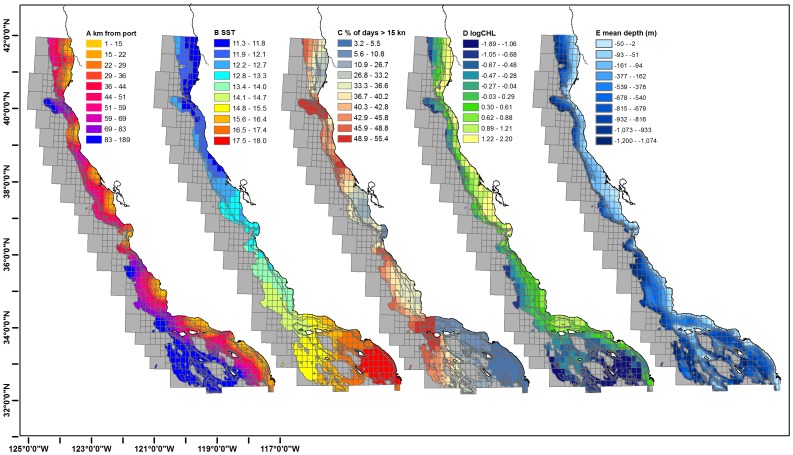
Potential explanatory covariates for GLMs by blocks. From left to right, 8a. distance (in kilometers) of the centroid of a given block to the nearest port. 8b. mean sea surface temperature (for the 1999–2009 period), 8c. percentage of days in which average winds in a given block exceed 15 kn/hour (for the 1999–2009 period), 8d. mean chlorophyll a levels (for the 1999–2009 period) and 8e. the mean depth of the habitat by block. Shown are the 100 m, 200 m, 400 m, 600 m, and 1200 m depth contours. Note that recreational fisheries only include 0–200 m blocks.

The environmental covariates for the California seascape demonstrate regional off-shore and latitudinal patterns (Figure 7a–e). Distance (km) from port is the greatest in the SCB, with the most distant blocks occurring up to 180 km from port. The ten-year average SST corresponds to a latitudinal gradient of colder water in the northern blocks and warmer waters in the southern blocks. The percent of days exceeding 15 knots is patchy, with calm winds in nearshore areas of the SCB, Half Moon Bay, between Eureka and Crescent City and within the Monterey Bay. High winds are located near Cape Mendocino, Point Arena, Big Sur, Point Conception and ‘outside’ the Channel Islands. Chlorophyll-a demonstrated a regional gradient with northern blocks having relatively high values of Chlorophyll-a, whereas southern blocks have medium to low Chlorophyll-a, with nearshore blocks having a higher value of Chlorophyll-a than offshore blocks. The mean depth of each block shows a general pattern of shallower water nearshore and deeper water further offshore.

**Figure 8:**
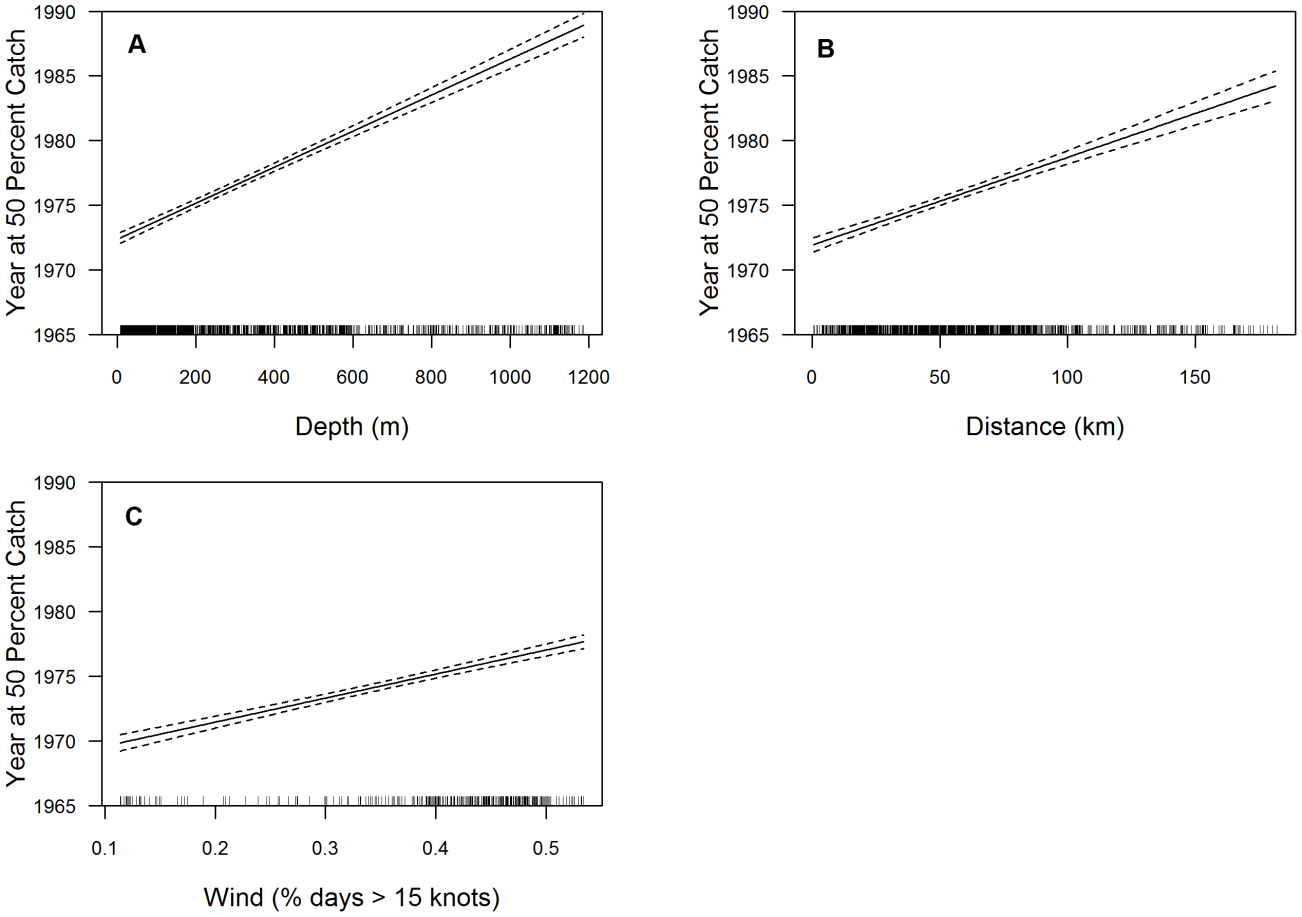
Generalized linear model fits for commercial groundfish. Where the y-axis is Year at 50% Catch (Yr50) and the x-axis is model variable, 8.a depth, 8.b distance from port, and 8.c wind. The dashed line indicates 95% confidence interval. Tick marks across the x-axis represent the distribution of observations.

**Figure 9:**
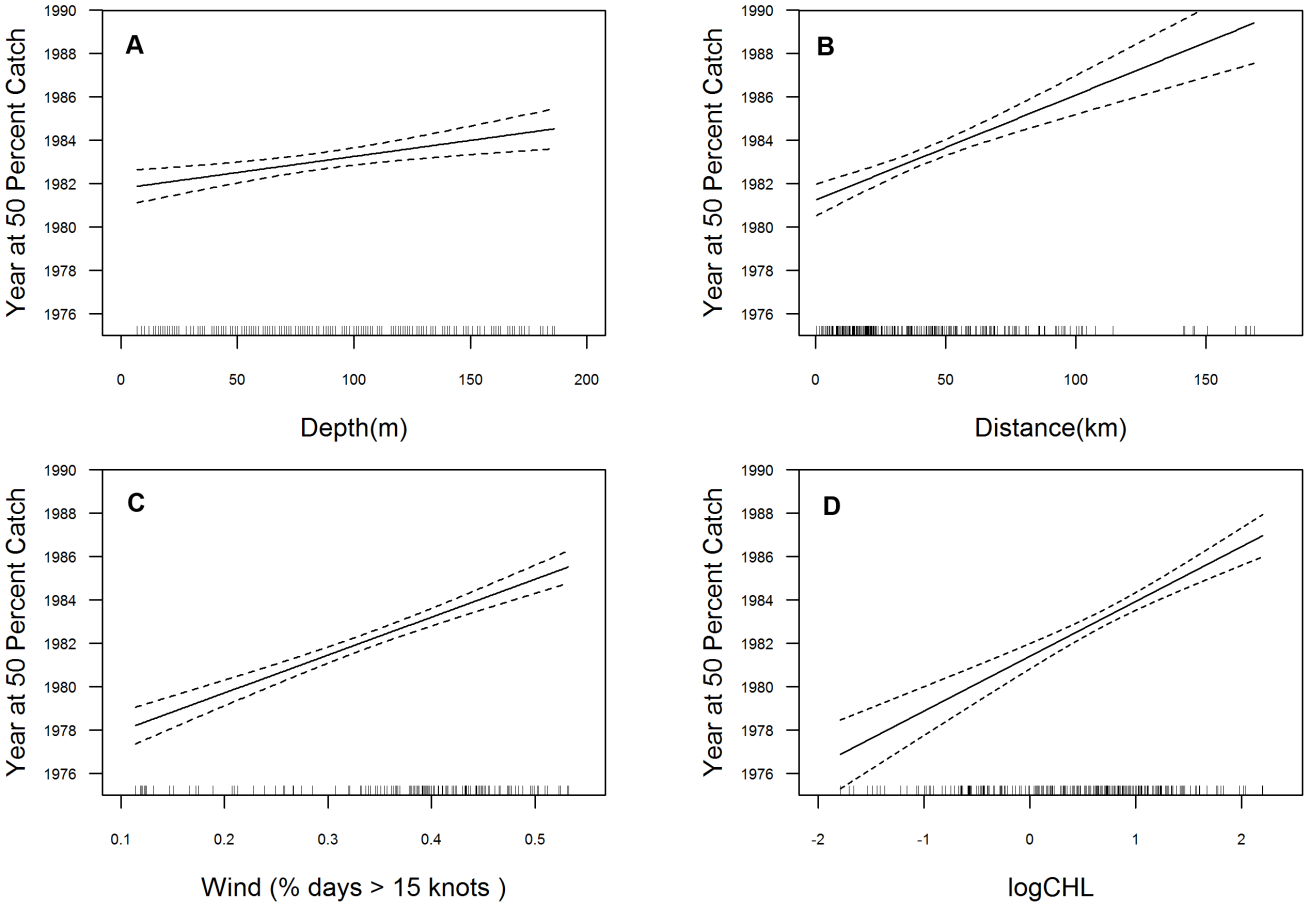
Generalized linear model fits for recreational rockfish. Where the y-axis is Year at 50% Catch (Yr50) and the x-axis is model variable, 9.a depth, 9.b distance from port, 9.c wind, and 9.d Chlorophyll-a. The dashed line indicates 95% GLM confidence intervals. Tick marks across the x-axis represent the distribution of observations.

The GLMs demonstrated that geographic expansion occurred for both commercial groundfish and recreational rockfish fisheries with a shift to deeper, more offshore habitat and more inclement weather conditions over time. The selected model for commercial groundfish Yr50 catch (Table 2) and associated plots show an increase in depth, distance from port and wind, demonstrating historical catches generally occurred earlier in shallower, nearshore habitat, closer to port, and in regions characterized by more favorable weather (less wind) (Figure 8a–c). For recreational rockfish Yr50, the selected model (Table 2) indicated wind, distance from port and logCHL had a steep positive slope, while depth demonstrated a moderate positive response with Yr50 (Figure 9a–d).

**Table 2:** Candidate generalized linear models representing Year at 50% Cumulative Catch (Yr50) for California commercial groundfish and recreational rockfish.

Fishery	Model	AICc	R2	weight
Commercial	**Depth, Wind, Distance**	**9434.4**	**0.31**	**1**
Recreational	**Wind, Distance, Depth, logCHL**	**1506.2**	**0.31**	**0.62**
Recreational	Wind, Distance, logCHL	1514.9	0.30	0.36

Modeling considered depth, distance from port, wind and chlorophyll-a (recreational only) as potential covariates. Presented are the candidate models where the weight was >0.00. The final selected model is shown in bold face.

**Figure 10:**
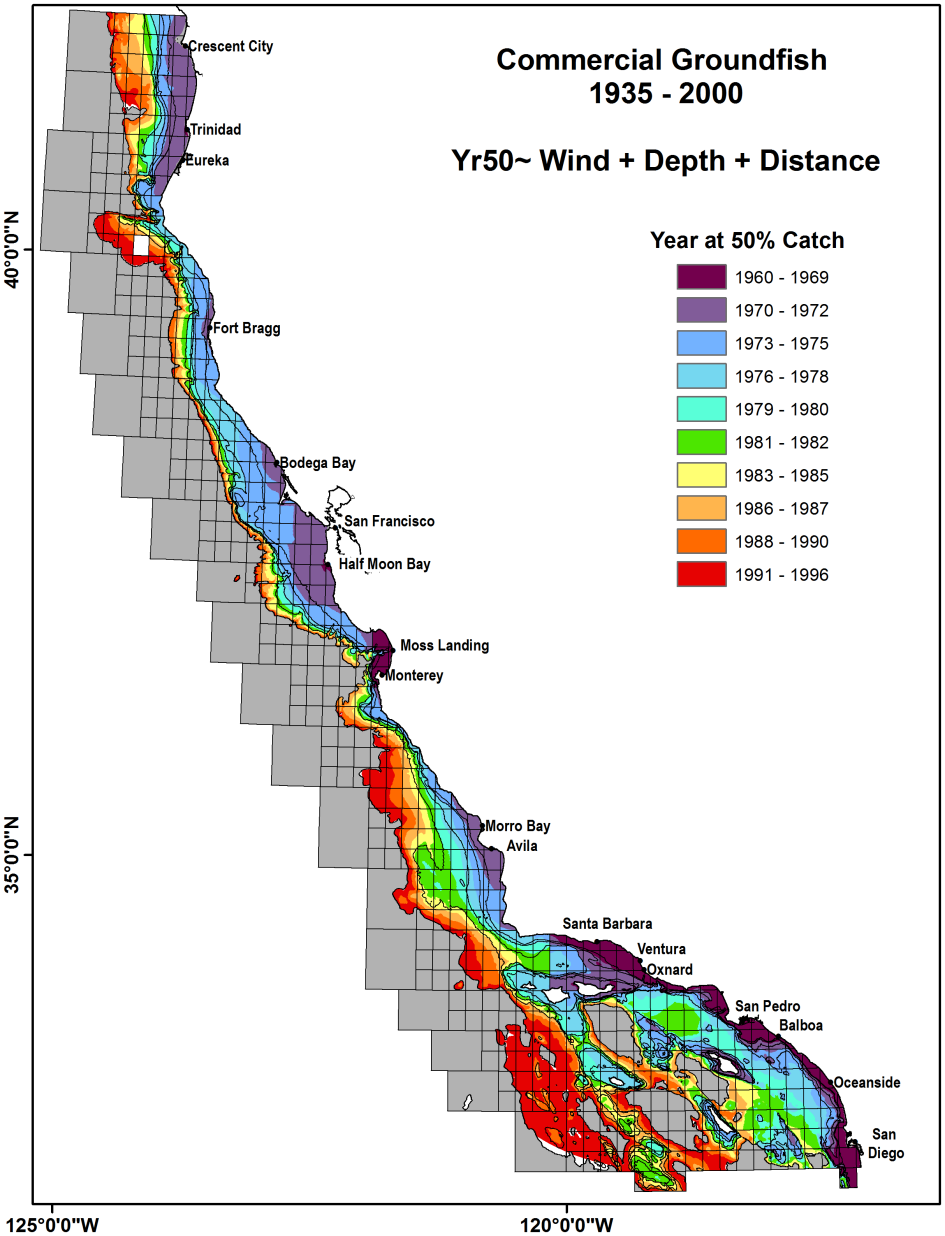
Modeled GLM for Year at 50% cumulative catch (Yr50) for commercial groundfish species. Modeled Yr50 was between the years 1960–1996. Classifications are based on an equal quantile distribution. Gridded overlay represents catch blocks stratified by depth strata representing habitat of each groundfish species, shown are 200 m, 400 m, 600 m, 1200 m. Ports shown are identified as the top 95% commercial ports by total catch.

**Figure 11:**
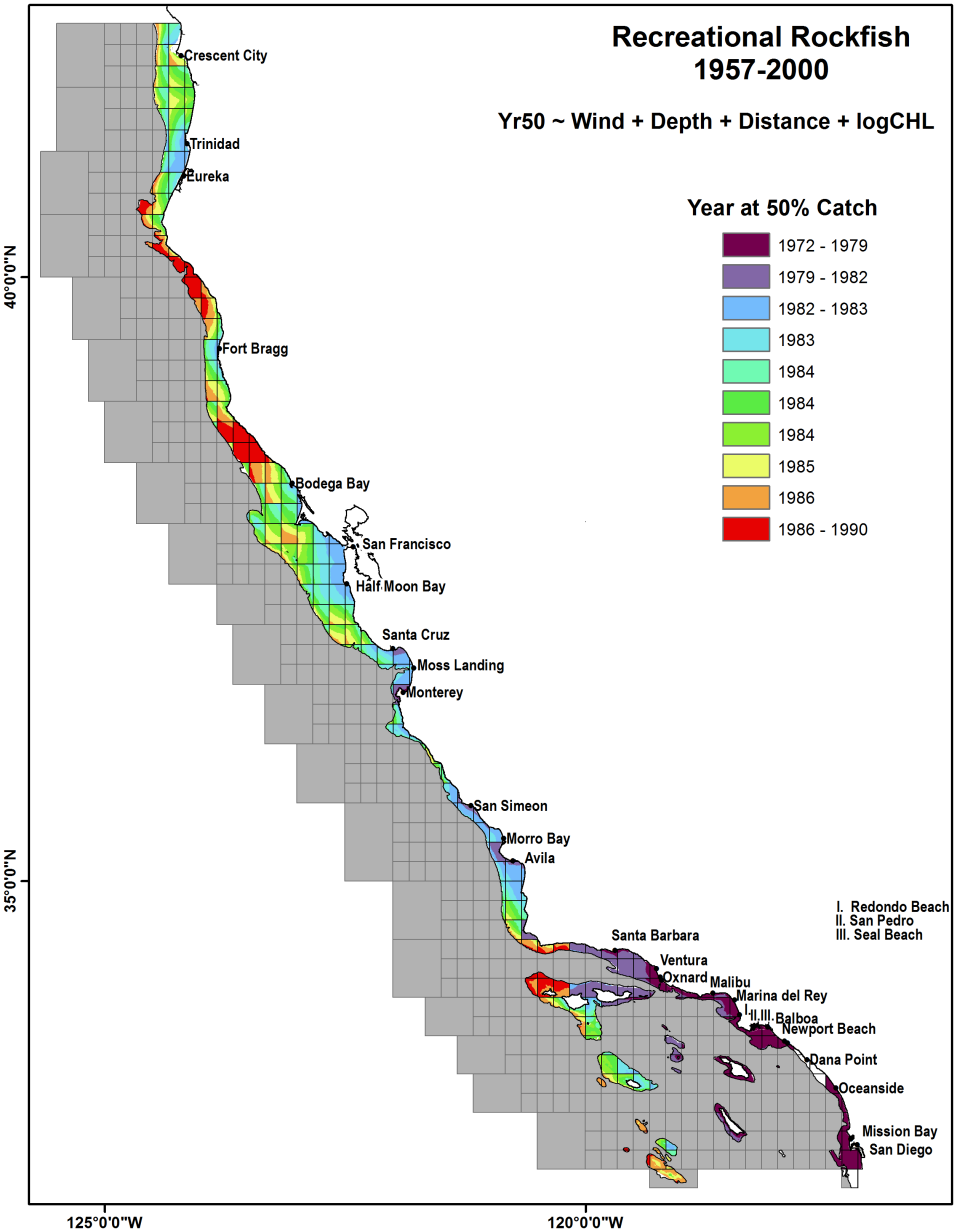
Modeled GLM for Year at 50% cumulative catch (Yr50) for recreational groundfish species. Modeled Yr50 was between the years 1973–1990. Classifications are based on an equal quantile distribution. Gridded overlay represents catch blocks stratified by 200 m representing fished habitat. Ports shown are identified as the top 95% recreational ports by total catch.

The modeled predictions for Yr50 catch for commercial groundfish demonstrated a nearshore/offshore gradient while recreational rockfish indicated a patchy regional latitudinal gradient (Figures 10 and 11). The commercial fisheries Yr50 modeled values ranged from 1960–1996 and occurred earlier near islands, historically active ports of Eureka, San Francisco, Monterey and the nearshore SCB. Yr50 occurred later for offshore, distant reefs such as Tanner/Cortez Bank in the SCB, the Santa Lucia Banks west of Avila and in the region near Cape Mendocino. Catches occurred later at deeper blocks, including deep blocks located just west of San Diego. The recreational fisheries Yr50 modeled values ranged from 1973–1989 and clearly show catch occurred earlier in the SCB (mid–1970s) and the nearshore Channel Islands (Anacapa, Santa Barbara and Catalina). Catch for Northern California recreational rockfish fisheries occurred in the 1980s with blocks just north and south of Fort Brag have Yr50 in the late 1980s.

## Discussion

Geographic expansion within the coastal waters of California was demonstrated for both commercial groundfish and recreational rockfish fisheries. The findings provide historical trends for fishing conditions experienced over the time period evaluated and the shift to other species and habitat blocks as fishery resources were exploited. The selection and movement to different blocks over the history of the fishery provides a proxy for the relative costs and benefits of fishing, where economic forces drive the expansion of fishing activity to more optimal habitats when other accessible habitat resources are fully utilized or depleted [1]. Our results show an increase in fishing depth during the modeled period for California groundfish. Consistent with our general observation is a historical comparison by Karpov et al. [71] who found that recreational rockfish catch shifted from typically shallow to deepwater species during the 1980s with an accompanying decline in average weight of the nearshore species. Movement towards more productive waters (Chlorophyll-a) increased over time for recreational rockfish, indicating a potential harvest shift to habitats where species experience more rapid life histories. In a comparison study in the SCB, rockfish occurring in more productive waters grew larger relative to age and had an earlier age at recruitment [72]. Wind and distance had a steep positive slope in our model response plots indicating that as time progresses, many fishermen have increased their willingness to fish more inclement weather and travel farther to available habitats in order to potentially catch slightly larger or greater numbers of rockfish. A former CPFV operator working out of Santa Barbara found similar long-term patterns using his own high resolution logbook data with respect to rockfish catch rates and the distance from port, depth, and climate covariates (M. McCrea, University of California Santa Barbara, unpublished data).

We used a novel approach combining historical satellite data and CDFW habitat grid-blocks to best represent a suite of factors that fishermen may consider before selecting areas to fish, however the some of the variables were correlated and we had to estimate the distance from port. Our Pearson’s analysis showed that the percentage of windy days was highly correlated to SST (commercial and recreational) and CHL was highly correlated to distance from to port (commercial). The strong couplings are expected because wind stress mixes the top layers of seawater thus influencing temperature while CHL is most abundant at narrow alongshore upwelling zones where the fishing ports are located [73]. For the GLMs, we included the percentage of windy days and distance from port because these factors would be more important considerations in day-to-day fishing operations than SST or CHL [74]. The study assigned the minimum Euclidian distance from the reported catch block to ports, due to the limited port information during the early years of the fishery, however it is possible that the real distances travelled to fishing grounds may be much greater than evaluated here as many fishermen would travel to and from their home port, rather than the nearest port.

Many of the species in this study were historically overexploited, and the maps generated suggest serial depletion may have occurred in some areas. It is evident that the commercial groundfish fishery selected the shallow 200 m species habitat blocks earlier and have more recently concentrated selection for deeper habitats and species. These deep water blocks were typically less intensively exploited for the time period evaluated. The Yr50 and CPA maps taken together may further provide evidence for serial depletion. For example, in areas where groundfish were caught early (Yr50 = purple or blue) and total cumulative catch is high (CPA = brown) (see Monterey Bay and nearshore northern California for commercial fisheries and nearshore SCB for recreational fisheries) it may be that more recent levels of effort in these habitat blocks is lower, potentially due to limited abundance of groundfish when compared to historical conditions. Although existing data cannot resolve within-species shifts in abundance and distribution, these patterns are consistent with many of the available stock assessment results for the species discussed here. For example, many shelf species of commercial significance, such as English Sole (*Parophrys vetulus)* and Petrale Sole, experienced high or peak landings, followed by significant declines in abundance by the 1940s and 1950s [75], [76], while more broadly but also more deeply distributed species, such as sablefish, Dover sole and thornyhead rockfishes have long exploitation histories but tended to experience peak landings and stock declines considerably later, as fisheries developed into deeper habitats [77]–[79].

The summarized CPA and plotted covariate response indicates that there may be spatial disaggregation between commercial groundfish and recreational rockfish resource extraction. Even though the historical recreational and commercial fisheries data did not allow for direct comparison of CPA (metric tons vs. # fish kept), a relative magnitude of catch can be compared. For recreational rockfish, a larger CPA value was demonstrated at inshore blocks located near ports, especially in central and southern California waters. A greater number of recreational ports with 95% of catch were geographically located in southern and central California, which agrees with the majority of the human population. In other regions of the world, fishing pressure increases with increasing population size [80]. In contrast, the CPA for commercial groundfish is further offshore and primarily located in central and northern California, where fewer ports and population is located. While the habitat selection over time increased in distance, depth and wind for both commercial and recreational fisheries, the plotted covariates for depth had a steeper slope in commercial fisheries than recreational fisheries, perhaps indicating that technological advances and new gear types to catch fish in newly available deeper habitats were important.

The legacy effects of geographic expansion may be evident in contemporary groundfish population abundance and size structures. Our maps demonstrate that SCB habitat within the Cowcod Conservation Area (CCA) had a different exploitation history than those habitats closer to ports. In the CCA, catch history (Yr50) from our modeled surfaces suggest fishing activity is more recent and the magnitude of fishing impacts are less (CPA), this is consistent with demographic studies which found larger, older, and more diverse fish occur in these offshore areas [27], [81]. In contrast, for SCB habitats closer to ports, the modeled historical catches (Yr50) occurred much earlier and fishing magnitude is relatively greater (CPA), such nearshore areas have been associated with an increase in the smaller, dwarf rockfish species complex [82]. Fishing legacy, represented here by Yr50 and CPA, may be an important variable for many fisheries demographic patterns and may be much more common than presently documented in other regions due to the lack of historical data.

This is the first effort to model geographic expansion of groundfish in California and will provide a better representation of the spatiotemporal catch reconstruction in stock assessments. Geographic expansion models are of great importance for species-of-concern stock assessments, such as the rockfish complex. Over 60 species in the rockfish genus occur in California waters, however the catch reconstruction dataset prior to 1969 lumped rockfish into 13 market categories, and individual species were not adequately recorded until the late 1970s and early 1980s. This inability to separate out rockfish species led to considerable challenges in estimating historical catches at the species level to support assessment and management activities [51]. Consequently, in the most recent catch reconstruction efforts, there was a potential bias towards overestimating historical catches of deep water species in the early years of the fishery, because deep water habitats were more likely to be targeted in the 1970s and 1980s than in the period from the 1930s to 1960s. Understanding the spatial patterns in which fisheries developed will improve species resolution of historical catches. The modeled historical catch will also improve the spatiotemporal accuracy of fish distributions for stock assessments, as well as surveys and habitat assessments which are presently based upon contemporary fish distribution patterns.

The geographic expansion model and recovered historical dataset provides resource managers and stakeholders an invaluable window to the historical context of California fisheries and interaction between users and the environment. Our results indicate that over the history of these fisheries, catches have taken place in areas of increasingly deeper habitat, with an increasing distance between catch locations and ports, and in more inclement weather conditions. The growing recognition of serial depletion and geographic expansion detected in the California Current ecosystem will contribute to efforts related to marine spatial planning, habitat impact assessments, quantification of ecosystem services, and ultimately to ecosystem-based approaches to management of marine fisheries and other resources [83]–[85]. The future of fisheries management will benefit from the appreciation of both historical and contemporary patterns of fisheries effort and catch.
